# Juvenile zebra finches learn the underlying structural regularities of their fathers’ song

**DOI:** 10.3389/fpsyg.2015.00571

**Published:** 2015-05-08

**Authors:** Otília Menyhart, Oren Kolodny, Michael H. Goldstein, Timothy J. DeVoogd, Shimon Edelman

**Affiliations:** ^1^Department of Psychology, Cornell University, Ithaca, NYUSA; ^2^MTA TTK Lendület Cancer Biomarker Research Group, BudapestHungary; ^3^Department of Zoology, Tel Aviv University, Tel AvivIsrael; ^4^Department of Biology, Stanford University, Stanford, CAUSA

**Keywords:** vocal development, zebra finch, birdsong, statistical learning, song structure

## Abstract

Natural behaviors, such as foraging, tool use, social interaction, birdsong, and language, exhibit branching sequential structure. Such structure should be learnable if it can be inferred from the statistics of early experience. We report that juvenile zebra finches learn such sequential structure in song. Song learning in finches has been extensively studied, and it is generally believed that young males acquire song by imitating tutors (Zann, 1996). Variability in the order of elements in an individual’s mature song occurs, but the degree to which variation in a zebra finch’s song follows statistical regularities has not been quantified, as it has typically been dismissed as production error (Sturdy et al., 1999). Allowing for the possibility that such variation in song is non-random and learnable, we applied a novel analytical approach, based on graph-structured finite-state grammars, to each individual’s full corpus of renditions of songs. This method does not assume syllable-level correspondence between individuals. We find that song variation can be described by probabilistic finite-state graph grammars that are individually distinct, and that the graphs of juveniles are more similar to those of their fathers than to those of other adult males. This grammatical learning is a new parallel between birdsong and language. Our method can be applied across species and contexts to analyze complex variable learned behaviors, as distinct as foraging, tool use, and language.

## Introduction

In altricial species developing individuals are often surrounded by a highly structured environment. Successful functioning among conspecifics requires appropriate responses to actions of others, such as the coordination of social turn taking between parents and toddlers (Pereira et al., 2008), or replying with a proper song type during avian territorial encounters (Beecher and Campbell, 2005). To use the structure provided by the rearing environment, developing cognitive systems must be able to recognize regularities, segment the continuous stream of information, and learn the underlying rules.

To compare statistical regularities in behaviors among multiple subjects, one needs computational tools capable of (i) detecting and describing the structure of behavior and (ii) comparing the results across individuals. When used together, these tools can reveal common patterns, quantify individual differences, and, for acquired behaviors, help elucidate the mechanisms of learning (see, e.g., Visser et al., 2007). We used two such tools – a group of models of grammar acquisition that is being developed for natural language applications (Nevill-Manning and Witten, 1997; Solan et al., 2005; Kolodny et al., 2014) and a family of procedures for quantifying graph similarity (Shokoufandeh et al., 2005; Pinto and Tagliolato, 2008; Wilson and Zhu, 2008) – to study the grammar-like structure of song in the zebra finch.

Zebra finch song is composed of syllables: complex sounds, separated by very short silence intervals. The process of song learning in the zebra finch is frequently described as imitation of the tutor’s song: according to the standard view, zebra finches develop by adulthood highly stereotyped songs, with a single “canonical” motif, which is the most frequently produced sequence of syllables (Zann, 1996; Brainard and Doupe, 2001), copied from their tutor. However, individuals do exhibit substantial diversity at the levels of syllable transitions (sequence linearity) and motif occurrences (sequence consistency; Scharff and Nottebohm, 1991), and about a third of motifs are non-canonical, including ones with syllable deletions, additions, or repetitions (Sturdy et al., 1999). Despite this diversity, zebra finch song has not been examined so far for the presence of probabilistic syntax-like patterns while using this full scope of variability in each individual’s song. Moreover, Lipkind et al. (2013) recently demonstrated that young zebra finch individuals can be made to switch their song structure from one syllable order to another, under an appropriate training schedule, and Lu and Vicario (2014) have shown that auditory patterns that reflect both adjacent and non-adjacent regularities are passively learned and encoded by zebra finches. Could natural song learning in the zebra finch include acquiring underlying structural regularities in song variation? If so, the task for a young learner is to distill statistical regularities from tutor songs and incorporate these into his own production. Any such statistical regularities in zebra finch song would indicate a new parallel between avian song learning and human language.

Furthermore, the distinction between structural or grammar-like regularities and those pertaining to the individual units comprising the vocalization sequence (“lexical” regularities) has not, to our knowledge, been attempted in any non-human species. (To appreciate this distinction, consider the sentences “This bird can sing” and “That pig will fly,” which are completely distinct lexically but identical in their grammatical structure.) If present, structural regularities in song would dramatically change our view of song learning, as probabilistic patterns shared by juveniles and their fathers would suggest a statistical learning mechanism and a complex, perhaps hierarchical, internal representation.

Our aim was to search for grammatical regularities in the full corpus of variation found across song renditions, both in temporal relations among syllables and in temporal relations among larger units, thus accounting for possible hierarchical structure. We then compared the regularities between fathers and sons across multiple families. If statistical learning plays an important role during song development, then statistically coherent patterns present in a tutor’s song should be reflected in the song of his offspring. These patterns should be more similar between fathers and sons than between unrelated males.

Using techniques devised for analyzing structure in human languages, we examined zebra finch song for evidence of hierarchical statistical regularities in song motifs and compared the resulting graph ‘grammars’ across individuals. By grammar we refer to the set of syntactic rules and principles by which song structures are created (cf. Soha and Marler, 2001). Typically in such projects, one uses the corpus of song recorded from an individual singer to infer a grammar for that individual (Nishikawa and Okanoya, 2006; Jin, 2009; Berwick et al., 2011; Jin and Kozhevnikov, 2011). Although corpora can be pooled across individuals, such pooling assumes that the same basic lexicon of units (syllables) underlies song production in all the individuals — a potentially problematic assumption, which is invalid in zebra finches in many cases. In contrast, we describe a computational method that transcends this limitation and makes no assumptions about the commensurability of the lexicons of different birds. It does so by quantifying graph similarity (graphs are network structures created on the basis of transition probabilities between syllables, as in finite-state grammars) — in ways that are purely structural and do not involve the labels (syllable symbols) that annotate the nodes (vertices) of the graphs.

**FIGURE 1:**
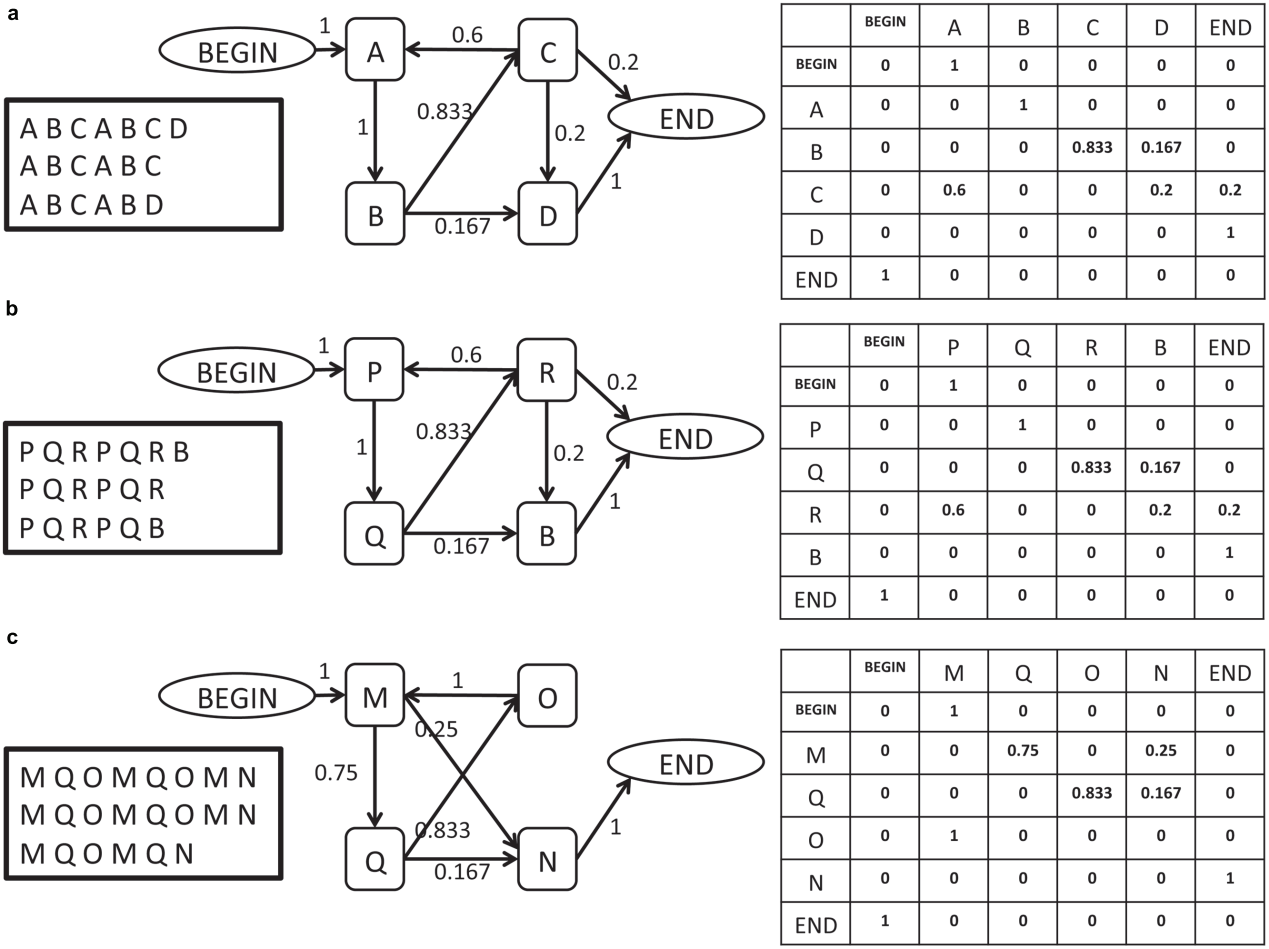
**Three illustrations of simple song corpora, representing the song of three birds (**a**, **b**, **c**), and their description as a SYL type grammar in the form of a graph and in the form of an adjacency matrix**. The BEGIN and END symbols are added by the grammar inference procedure. Each element *e_i,j_* in the matrix represents the weight of the edge that links syllable *i* to syllable *j* in the grammar.

**Figure 1** provides an intuitive illustration of some of the challenges that the proposed method is designed to overcome (full details of the methods are found below) and of the manner in which the data are analyzed for this purpose. The figure illustrates in its leftmost panel three short song corpora produced by birds (a), (b), and (c). Each row of letters represents a song bout, and each letter represents a syllable. Notably, each bird has a different repertoire of syllables from which its songs are composed, with very partial overlap of syllable sets between birds. Interestingly, even this partial overlap may be misleading: birds (a) and (b) share a common syllable, represented by *B*, but its role in their songs is very different: in bird (a), syllable *B* is part of a recurring sequence, *A B C*, that may be viewed as the canonical motif, while in bird (b) it is not. Moreover: it seems that syllable *B* plays a similar role in the song of bird (b) as syllable *D* does in the song of bird (a), a similarity that may be missed if one assumes that acoustically similar syllables in different birds are analogous to one another.

These examples illustrate the need for a comparison method that does not require repertoire (lexicon) commensurability among the birds whose songs are being compared. To the right of each corpus in **Figure 1**, there is a graph representation of a finite-state grammar that describes the song corpus, whose vertices are the basic syllables. To the right of that, there is an adjacency matrix representation of the graph (see below for definitions and details). In this simple illustration one can readily notice that the grammars of birds (a) and (b) are identical in their structure, reflected both in the existence/non-existence of edges between the vertices of the graph and in the weights of the existing links. Crucially, this comparison is possible despite the lexical incommensurability of the songs of these two birds. Moving on to the graph representation of the song of bird (c), one notes that despite some visual similarity to birds (a) and (b) in the song corpora and in the general layout of their graph representations, there are significant differences between them. These are reflected in the edge structure of the graph and in the edge weights. This illustrates that two grammars can differ in their structure in various ways, which makes the task of quantifying the distance between them non-trivial.

## Materials and Methods

### Subjects

Nine established zebra finch pairs were set up in single cages with a nest box and nest material, in a community room in both visual and acoustic contact with each other and with birds in other aviaries. The birds were kept on a 14:10 light cycle and were provided with finch seed and water *ad libitum*. Each pair nested, laid eggs, hatched and fledged 2–5 young (mean = 4.0 ± 1.2). Families were kept together for more than 3 months (99 ± 9) days. The juveniles were then transferred to single sex aviaries with other juvenile birds. The number of sons in each of the nine families was, respectively, {1,1,1,2,2,2,2,3,4}, leading to a mean and standard error of 2 ± 0.33.

### Recordings

Songs of all nine fathers were recorded. Mature female-directed song was recorded from 15 sons at the age of 108 ± 14 days to ensure ecological realism and relevance with regard to song production. Songs from three additional sons was recorded at the age of 142–153 days. For all recordings, males were placed in a sound proof room overnight in a 46*44*36 cm cage. The following morning, an adult female zebra finch was placed in an identical cage next to the male’s cage and recording began. If the male did not sing within 60 min, further recordings were attempted after a day back with the colony, until we obtained at least 10 song motifs in a single recording. The number of song bouts and the total number of syllables annotated for each individual is included in **Table 1**. All recordings used a Sennheiser shotgun microphone attached to a Canon MiniDV ZR930 camcorder on Fujifilm DVCassette miniDVs. MiniDV tapes were digitized with a JVC Super VHS ET Professional deck at 44.1 KHz. Uncompressed sound files were created using Soundtrack Pro 6 and were saved as separate wav files.

**Table 1:** The number of song bouts and overall number of syllables produced by each subject in the dataset.

Family	Subject	Number of song bouts	Number of syllables
1	302	32	458
1	410	30	942
1	303	10	192
2	427	77	1286
2	423	19	188
2	416	4	64
2	424	193	2607
2	625	17	272
3	428	6	145
3	422	6	226
3	624	83	1032
4	429	129	1381
4	627	82	1263
5	601	153	2467
5	527	203	2660
5	528	200	2220
5	707	33	490
6	629	81	941
6	610	29	170
6	612	22	186
7	619	259	3331
7	715	42	719
8	628	46	632
8	713	29	208
9	709	120	1247
9	708	168	1356
9	716	11	185

*A 1 h long recording of each individual was used.*

### Song Annotations

**FIGURE 2:**
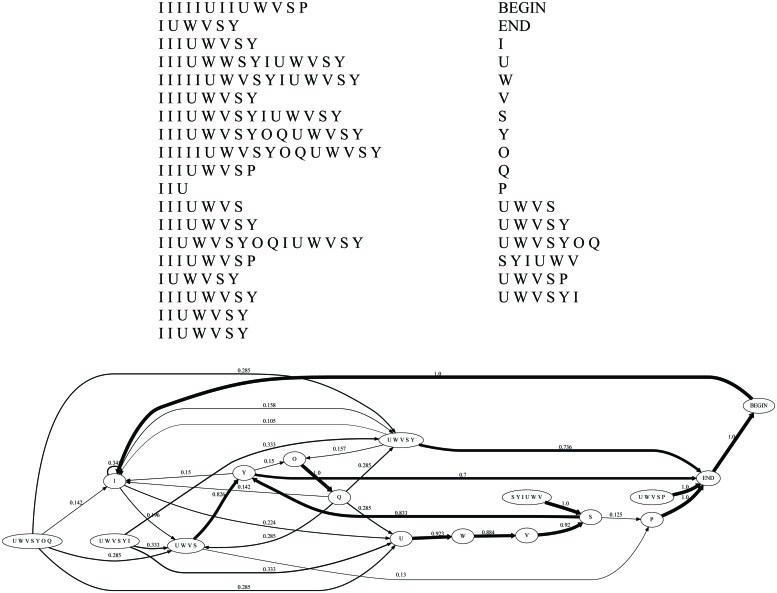
**Corpus, lexicon, and grammar of a single individual (#423)**. Clockwise from left top: the entire *corpus* (each line represents a song bout); the *lexicon* (syllables + motifs) for the COL type grammar without introductory notes (the BEGIN and END symbols are added by the grammar inference procedure); and the COL type *grammar* inferred by our model.

A song bout consists of some repetitions of a single note (*introductory notes*; see Price, 1979) followed by one or more song motifs (Price, 1979; Sossinka and Böhner, 1980). Song bouts in our data set were defined as strings of syllables in which all silent intervals were shorter than 500 ms, and every uninterrupted sound was defined as a separate syllable (cf. Williams, 2004). A song bout typically included 2–10 repetitions of the introductory note and 1–8 repetitions of a motif (Price, 1979). Because the present research investigates probabilistic dependencies among syllables combined into stable sequences or motifs, we denoted each syllable type in each individual’s song by a letter (Price, 1979; Eales, 1985). Every song in the recordings was then broken down into these constituent syllables and transcribed as a sequence of letters using the Syrinx software (John Burt, www.syrinxpc.com). See **Figure 2** for an example of the full corpus of songs from a single individual.

### Inferring the Grammar

For each individual’s song corpus, we derived four types of finite-state grammar using a variant of a biologically inspired model of language acquisition, U-MILA (Kolodny et al., 2014), and two grammars using previously proposed language acquisition models, ADIOS (Solan et al., 2005) and SEQUITUR (Nevill-Manning and Witten, 1997).

All grammars had the form of a probabilistic first-order Markov graph specifying the transition probabilities among basic units. The grammars differed in the choice of units that formed the lexicon, and hence in their ability to capture hierarchical structure. The basic building block of the lexicon units in all cases was the syllable. The four grammars derived by U-MILA were based on syllables only (SYL), collocations of syllables (COL), long recurring sequences only (MOTIF), or syllables accompanied by the most frequent recurring sequence in the corpus (SYL+M1). For the first type of grammar, SYL, the units used in learning of the Markov model were only the actual syllables. For the second type, COL, collocations of syllables forming longer units were allowed as well. We used the concept of collocation, borrowed from computational and corpus linguistics (e.g., Mel’čuk, 1998; Croft, 2001; Arnon and Snider, 2010), to operationalize the idea of “motifs” found in the behavioral literature on birdsong analysis (Sturdy et al., 1999; Brainard and Doupe, 2001). Intuitively, a collocation is a sequence of basic units that recurs in a corpus more often than warranted by chance.

From the normative computational standpoint, the search for significant collocations may follow the minimum description length (MDL) principle (Rissanen, 1987), which has been proposed as a method for grammar acquisition (Grunwald, 1994) and used with great effect for learning natural language morphology (Goldsmith, 2001). ADIOS (Solan et al., 2005) and SEQUITUR (Nevill-Manning and Witten, 1997) have been shown to constitute approximations of such an approach; in U-MILA, for the purposes of the present study, we approximated the MDL approach (which can be computationally problematic; see Adriaans and Vitanyi, 2007) by a heuristic greedy search procedure.

Specifically, in the COL grammar our model identified motifs with recurring sequences of syllables in each song corpus. Only sequences that did not contain an inner repetition of more than two syllables and that did not end in a partial repetition of their own first syllable/s were added to the lexicon (e.g., not retained: “a b c d a b c d,” “a b c d a b”; retained: “a b c d”). Among these, only sequences that occurred more frequently than a certain threshold were added to the lexicon, which also included by default all single syllables. A range of different parameter values in the search for such sequences led to similarly significant results.

The third type of grammar among those listed earlier, MOTIF, is similar to COL, but differs in that it ignores occurrences of syllable sequences that are sub-sequences of longer units that adhere to the limitations described above. Thus, MOTIF is more stringent than COL in its choice of units, leading to a smaller number of chosen units and to units that are on average longer than those in COL, and generally in line with the sequences typically viewed by researchers as proper motifs and non-canonical motifs.

Lastly, the SYL+M1 grammar is simply the set of separate syllables in the corpus, accompanied by the most frequent among the sequences chosen by MOTIF.

All grammars were explored in two modes: one that allowed introductory notes to be a part of a unit’s sequence, and one in which introductory notes were eliminated from the units’ sequences.

As an illustration, **Figure 2** shows the song corpus of one of the birds, along with the lexicon (syllables + motifs) for the COL type grammar without introductory notes, and the actual grammar inferred by our model.

Each of the four grammars included in our comparison reflects a different stance with regard to the question of what is the meaningful unit in zebra finch song. SYL is the simplest of the four: it does not represent high-order units as such, implicitly assuming that the regularities that govern song production are defined exclusively over the basic elements. Since it contains all (and only) the syllables that comprise the bird’s song corpus, it offers the minimal framework for describing the full corpus in the form of a first-order Markov graph. In this sense, the three other grammars are extensions of SYL, each containing in its representation the individual syllables as well as some additional higher-order sequences. The COL and MOTIF grammars assume that songs are composed of large ‘chunks’ that are combined in various ways to give rise to the full song variability. COL is permissive, allowing a wide range of sequences, with few prior constraints, to serve as chunks, while MOTIF is more stringent, with the higher-order units that it allows being subject to a set of requirements. These requirements are intended, as noted above, to make the emerging repertoire include what is typically described in birdsong literature as ‘non-canonical motifs’ alongside the canonical motif. The SYL+M1 grammar allows only a single high-order unit, the canonical motif, and can be interpreted as assuming that the song is essentially composed of a single high-order unit and potentially some local (non-random) deviations from it, which together give rise to the observed variability in the song. Importantly, an approach that *a priori* dismisses all digressions from the canonical motif as random production errors is incompatible with our present effort, because it eliminates from the collected data any variability and with it the potential for uncovering grammatical regularities beyond that of a single linear sequence.

It is *a priori* unclear which grammar from among the above is the most appropriate one for describing zebra finch song. As a theoretically motivated approach to this question, we used a leave-one-out (LOO) cross-validation procedure to select, among the different types of grammars produced by U-MILA, the one that assigns the highest probability to a withheld portion of the song corpus for each individual. For each such song corpus and for each grammar type, we repeatedly set aside a single song (iterating eventually over each song in the corpus), trained the model on the remaining corpus, and used the resulting grammar to estimate the probability of the withheld song.

**FIGURE 3:**
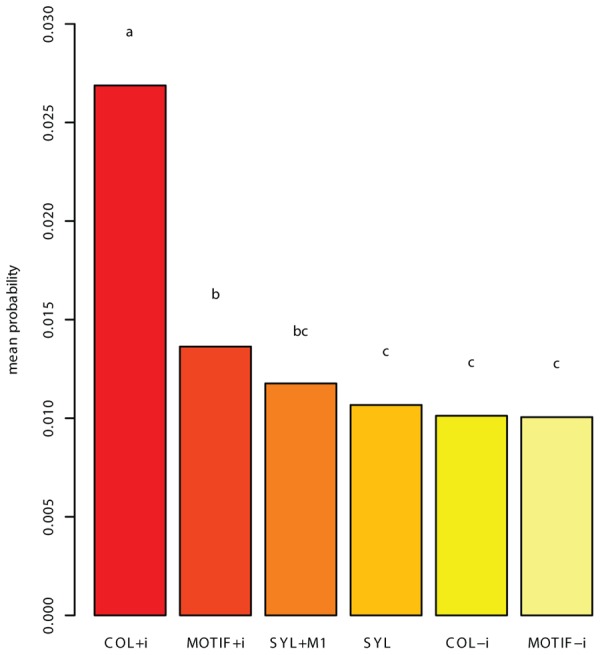
**The mean probability assigned to a song produced by a bird, after training on the rest of that bird’s corpus**. The result presented is a mean, per grammar, of means calculated per bird. The letters above the bars signify groups: bars marked with the same letter do not significantly differ according to the Tukey HSD test, with *p* < 0.0001. Grammar COL+i (COL, allowing introductory notes in units) assigns a significantly higher mean probability to the withheld test songs than all other grammars, and is accordingly the grammar on which the subsequent analysis focused.

The means of the resulting probabilities over the 27 birds were computed for each competing grammar. These scores served as the basis for two tests. First, the mean unseen song probability was calculated for each grammar. This measure revealed the COL grammar that included introductory notes (COL+i) as significantly more successful than the other grammars, among which the differences were smaller (**Figure 3**). Second, we conducted a binomial test that counted, for each pair of grammars, the number of birds for which the first grammar’s mean score was greater than the other’s. In this test too, the COL+i grammar came out as significantly better-performing than all others. Additionally, we found that SYL and SYL+M1 scored higher than MOTIF-i (MOTIF without introductory notes) for a significant majority of birds, and that MOTIF+i did the same compared to COL-i.

The finding that in zebra finch song COL+i is more appropriate than the other candidate grammars that we considered, including, in particular, COL-i, suggests that in analyzing a bird’s song it is prudent to avoid dismissing out of hand parts of the song corpus such as the introductory notes and any other potential sources of non-random and perhaps meaningful variability. The relative success that COL+i and MOTIF+i had in accounting for the data compared to COL-i and MOTIF-i, which leave out introductory notes, suggests that there are important regularities in the number of introductory notes that initiate each song bout. Having found that the COL+i grammar was the most successful in describing the birds’ songs using the two measures mentioned above, we focused on this grammar in our subsequent analysis. Yet, since all grammars described above have the capability of capturing structural aspects of the song, we applied our analysis to the other grammars as well, to find out whether or not our findings are robust to the specific choice of grammar.

### Estimating Similarity of Grammars

Multiple techniques exist for comparing graphs. The main constraint on the choice of graph similarity in the present case was the decision to avoid using vertex labels (due to the possible incommensurability of individual lexicons). This rules out the use of obvious measures such as graph edit distance, in which the dissimilarity between two graphs is defined as the smallest number of vertex and edge deletions, insertions, and substitutions that transform one graph into the other. The most straightforward remaining option is spectral graph distance, defined as the Euclidean distance between the lists of eigenvalues of the adjacency matrices of the two graphs (e.g., Wilson and Zhu, 2008). The two lists of eigenvalues are sorted in decreasing order; if one of them is shorter than the other (because the adjacency matrix has a lower rank), it is padded with zeros. In the present case of graphs corresponding to probabilistic finite-state grammars, the structure of the graph is properly described by a real-valued transition probability matrix (rather than a binary one), which is not necessarily symmetric. The eigenvalue spectrum of such a matrix is generally complex-valued. Accordingly, the spectral distance is defined as the Euclidean distance between ranked absolute values of the complex eigenvalues (Shokoufandeh et al., 2005; Pinto and Tagliolato, 2008).

This spectral distance, which we refer to as Spectral, and which for binary adjacency matrices is known to closely track the edit distance (Wilson and Zhu, 2008), while avoiding any use of vertex labels, is well-suited *a priori* to the task of comparing song grammars, where deletions, insertions, and substitutions of elements are the most natural causes of song difference. We focused on this measure of similarity in the present exploration. We also performed our analysis using two related measures, one based on the eigenvalue spectrum of the symmetrized (undirected) weighted adjacency matrix, obtained by summing the transition probability matrix and its transpose (WSpecAdj), and the other on the eigenvalue spectrum of the (undirected) binary adjacency matrix, obtained by replacing non-zero real-valued weights in the weighted adjacency matrix with 1’s (SpecAdj).

The spectral distance was chosen for the main analysis because among the candidate measures it is the only one that takes into account the weight of links, i.e., the real values that correspond to the transition probabilities between the units over which the grammar is defined – a characteristic that is perhaps the most informative one when comparing graphs in which a large proportion of the possible edges are in place.

#### Additional Measures of Graph Distance

In addition to the three measures of graph distance described, we explored two other measures. The first is SpecNormLap, which is defined in terms of the eigenvalues of the normalized graph Laplacian. The Laplacian is computed from the graph’s symmetrized (undirected) adjacency matrix (the Laplacian of a graph is defined as the difference between its degree matrix and its adjacency matrix; see Wilson and Zhu, 2008 for details). Because this distance measure is known to afford a finer discrimination between similar graphs, we expected it to be less useful for the present purposes — quantifying song relatedness, not distinctions — than those described in the main text.

The second of the two additional measures of distance between graphs, CNAFeat, is based on a family of graph features used in computational network analysis (CNA); the particular features we considered have been used for characterizing brain dynamics and are part of the Brain Connectivity Toolbox (BCT; Rubinov and Sporns, 2010). Because of the diverse nature of these features, some of which are global (pertain to the entire graph) and others local (per-vertex), we employed the Mahalanobis distance, which weights individual dimensions by their variance. The composition of the graph feature vectors (ordered lists of features) that we looked at is as follows (for definitions of each measure see Rubinov and Sporns, 2010): transitivity (global); clustering coefficient (per vertex); modularity index (global) and module membership (per vertex); betweenness centrality (per vertex); 3-vertex motif intensities for the 13 classical motifs (per vertex); and 4-vertex motif intensities for the 199 classical motifs (per vertex).

Neither of these two measures yielded significant differences between SAME and DIFF grammar pairs when calculated using the SYL and COL grammar types. Note that the second measure is a representative of a large family of measures which may be composed of the features we utilized and of others, using different weighing schemes and focusing on any of the numerous characteristics of graphs. Further exploration of such distance measures may be fruitful.

### Statistical Analysis

While juvenile birds mainly learn their father’s song (Zann, 1996), there are indications of horizontal song transmission among male siblings (Derégnaucourt and Gahr, 2013), and sibling interactions influence learning outcome (Tchernichovski and Nottebohm, 1998). We therefore grouped birds based on whether they belonged to the same or to a different family. Data from the 27 birds gave rise to 27*(27-1)/2 = 351 possible pairwise comparisons; of those, 31 pairs were defined as SAME-family (father–son or siblings) and the remaining 320 pairs as DIFFerent (unrelated) for the purposes of the analysis. Our dependent variable was the similarity between grammars. To avoid relying on assumptions of normality, equal variance, etc., we employed a non-parametric test, the Kruskal–Wallis statistic, to estimate the significance of the difference between the similarity values in the SAME and DIFF conditions. This test was performed for each of the grammar types, using each of the measure of graphs’ distance described above. In addition, for each of these cases, we conducted 31 Wilcoxon one-sample sign rank tests, each comparing the value of grammar similarity for one of the SAME pairs to the list of values of all 320 DIFF pairs. Each such test was performed with alpha = 0.05/31 = 0.0016, which incorporates the Bonferroni correction for multiple comparisons. Finally, with S denoting the number of those tests that came out as significant, we conducted a binomial test of the significance of having S successes out of 31 trials.

## Results

We compared songs of individuals within and across families. A syllable catalog was created for each male by assigning a symbol to each syllable type. Samples of each bird’s song were subsequently annotated using this catalog and processed so as to yield a graph-structured grammar. We described each male’s song by finite-state grammars of several types, all defined by transition probability matrices among units: syllables, or sequences of syllables. The grammars differ in their choice of units (see Materials and Methods). To assess similarity in the grammar structure of juveniles and fathers, the grammars of individuals were compared pairwise, distinguishing within-family and between-family pairs of comparisons (SAME and DIFF). For comparison purposes, we represented each grammar by the eigenvalue spectrum of its transition matrix (the “Spectral” measure; see Materials and Methods). This method is correlated with graph edit distance (the number of steps needed to transform one graph to another; Wilson and Zhu, 2008).

**FIGURE 4:**
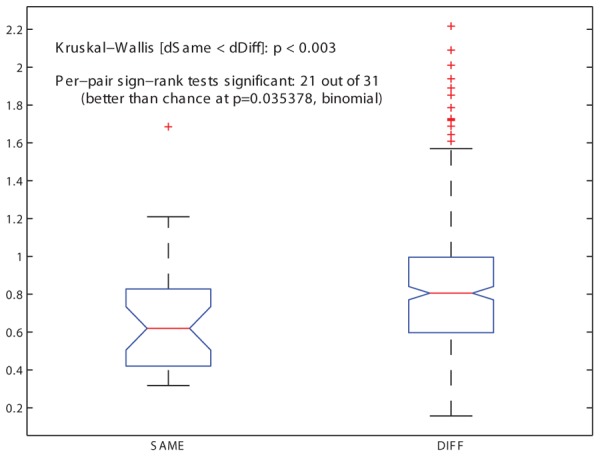
**Similarity among pairs of song grammars**. The distribution of grammar similarity values for the 31 pairs of related individuals (SAME family) and for the 320 pairs of unrelated ones (DIFFerent families), for the COL grammar type (based on syllables + motifs) and the Spectral grammar similarity measure (based on the eigenvalue spectrum of the matrix), showing medians, first and third quartiles (box), limits of 1.5 times the inter-quartile range (whiskers) and outliers (+ symbols), where higher values indicate greater distance between grammars and thus lower similarity. The median of the SAME distribution is significantly lower than that of the DIFF distribution (*p* < 0.003, Kruskal–Wallis rank sum test), indicating greater similarity in songs of related individuals. Of the 31 pairwise similarity values for SAME birds, 21 were significantly lower than the median similarity value for DIFF birds (Wilcoxon sign rank test with Bonferroni-corrected alpha of 0.0016). A binomial test showed this pattern to be significant (*p* < 0.036).

A LOO cross-validation process, in which each grammar was tested for its ability to accommodate a previously unseen song that had been omitted from the training corpus, found that one of the grammars, COL, is significantly more successful than the others in describing the withheld song (see Materials and Methods and **Figure 3**). Comparing grammars of this type for related and unrelated individuals yielded the predicted statistical regularities: the mean grammar similarity under the Spectral distance measure between related males was greater than the mean similarity between unrelated males (**Figure 4**). A Kruskal–Wallis test revealed this difference to be highly significant (*p* < 0.003). A binomial test for Bonferroni-corrected significant pairwise outcomes (21 out of 31) was also significant (*p* < 0.035; see Materials and Methods). These findings held also for a version of the COL grammar in which units in the grammar were precluded from containing introductory notes (cf. Tchernichovski and Nottebohm, 1998): the mean distance between related males’ grammars was smaller than that of unrelated males’ grammars (*p* < 0.005), and the binomial test yielded identical results to the COL grammar that included introductory notes.

To make sure that the significant result that we find is not generated by unusually high song similarity within a particular family, we repeated the analysis with eight of the nine families, excluding a different family every time, using the COL grammar. In all nine analyses that this gives rise to, the mean grammar similarity between related males was according to the Kruskal–Wallis test significantly greater than the mean grammar similarity between unrelated males.

We validated this basic result further by performing a bootstrap analysis (Efron and Tibshirani, 1993), in which both the SAME/DIFF similarity comparison and the binomial test were carried out on randomized versions of the transition matrices, obtained by pairwise reshuffling of non-zero weights in the original matrices learned by the model with the COL grammar (we performed this test also on randomly column-reshuffled data, with the same outcome). The randomization and the tests were repeated 1000 times, yielding an estimate for the standard deviation of each of the statistics considered in the original analysis. As expected, the similarity values derived from the randomized data were statistically indistinguishable between SAME and DIFF conditions; likewise, the binomial test on randomized data yielded a number far below the one for actual data [13.79 ± 2.14 (mean and SD) out of 31, compared to 21 out of 31].

To explore the robustness of our findings, we conducted the same analyses for multiple grammars and grammar similarity measures, including those that were less successful than COL in the cross-validation procedure and including measures that seemed less suitable than Spectral for revealing structural regularities (for details, see Materials and Methods and **Table 2**). A number of insights from these explorations are offered below.

**Table 2:** Comparison of grammar similarity between related (SAME) and unrelated (DIFF) birds.

	Spectral					Weighted AdjSpect					Unweighted AdjSpect				
	Kruskal–Wallis			Binomial per-pair		Kruskal–Wallis			Binomial per-pair		Kruskal–Wallis			Binomial per-pair	
Grammar	SAME	DIFF	*P*-val	Significant comparisons	*P*-val	SAME	DIFF	*P*-val	Significant comparisons	*P*-val	SAME	DIFF	*P*-val	Significant comparisons	*P*-val
SYL	0.69	0.77	0.17	16	0.5	0.5	0.58	0.12	18	0.24	1.98	2.35	0.034	17 of 31	0.36
COL+i	0.68	0.85	0.003	21	0.035	0.62	0.72	0.07	19	0.14	6.08	6.99	0.078	21 of 31	0.035
COL-i	0.67	0.83	0.005	21	0.035	0.6	0.71	0.044	18	0.24	5.39	6.23	0.19	20 of 31	0.07
MOTIF+i	0.69	0.83	0.008	21	0.035	0.5	0.61	0.008	23	0.005	2.64	2.77	0.49	16 of 31	0.5
MOTIF-i	0.71	0.83	0.02	21	0.035	0.52	0.61	0.016	21	0.035	2.41	2.79	0.05	17 of 31	0.36
SYL+M1	0.71	0.8	0.12	18	0.24	0.52	0.6	0.028	20	0.075	2.07	2.36	0.12	17 of 31	0.36
ADIOS+i	0.7	0.86	0.003	22	0.015	0.67	0.8	0.031	17	0.36	8.64	9.11	0.79	14 of 31	0.76
ADIOS-i	0.7	0.85	0.016	21	0.035	0.64	0.8	0.003	22	0.01	5.87	6.18	0.83	17 of 31	0.36
SEQUITUR+i	0.87	1	0.148	16	0.5	1.04	1.26	0.09	19	0.14	15.23	16.99	0.49	19 of 31	0.14
SEQUITUR-i	0.87	1.02	0.065	18	0.24	1.06	1.3	0.036	20	0.07	10.17	11.81	0.15	19 of 31	0.14

*The table summarizes all comparisons among all pairs of birds, using 10 types of grammar and three measures of distance among grammars, where higher values indicate greater distance between grammars and thus lower similarity. The comparison among SAME and DIFF pairs of birds for each (grammar + measure) combination was subjected to a Kruskal–Wallis test and an independent Binomial test. *p*-values reported above refer to the significance of a difference between SAME and DIFF pairs of grammars. Rows with *p*-values below 0.05, suggesting that grammars of birds within the SAME family are significantly more similar in their structure than grammars of birds from DIFFerent families, are highlighted.*

(1) The grammar that we called SYL, which does not allow hierarchies and whose units are the original syllables in the bird’s repertoire, and the grammar called SYL+M1, which in addition contains as a unit the single most frequent recurring sequence in the bird’s corpus (usually termed dominant motif), were both less successful than COL in accounting for previously unseen songs (see the cross-validation process in the Methods, and **Figure 3**). This finding is of interest, as these are the two common ways in which zebra finch song is described (e.g., Zann, 1993; Sturdy et al., 1999). Neither of these two grammar types yielded a significant difference between SAME and DIFF pairs under the Spectral measure.
(2) The grammar that we called MOTIF, whose units are the bird’s original syllables along with a number of long recurring sequences (aimed to cover both what is generally referred to as dominant motif and non-canonical motifs), had similar success in the cross-validation procedure as did SYL and SYL+M1, but did show a significant difference between SAME and DIFF pairs, both when introductory notes were included and when omitted.
(2) We also analyzed the birds’ song using grammars designed to represent human language in a compact manner, ADIOS (Solan et al., 2005) and SEQUITUR (Nevill-Manning and Witten, 1997). The SAME-DIFF pairs comparison using the former showed significant differences both with introductory notes included and without them; for the latter, this comparison yielded no significant differences. The success of the ADIOS-derived grammar in exposing significant differences suggests that the results we report above are quite robust: while the conceptual approaches underlying ADIOS and U-MILA are related, the algorithms they use are quite different. Clearly, however, the details of the approach matter, as suggested by the negative outcome with SEQUITUR.
(4) We repeated the analyses with two additional measures of grammar distance that are similar to Spectral: one based on the eigenvalue spectrum of the symmetrized (undirected) weighted adjacency matrix (WSpecAdj), and the other on the eigenvalue spectrum of the (undirected) binary adjacency matrix (SpecAdj; see Materials and Methods). The former successfully uncovered structural similarities in SAME pairs compared to DIFF pairs for a number of the grammars described above (SYL+M1, COL without introductory notes, MOTIF with and without introductory notes, ADIOS with and without introductory notes, SEQUITUR without introductory notes), while the latter showed a marginally significant difference only with two grammars (SYL, MOTIF without introductory notes). This finding indicates that critical information regarding the structural regularities of a grammar resides in the strength of links among units, and not only in those links’ presence or absence.

**FIGURE 5:**
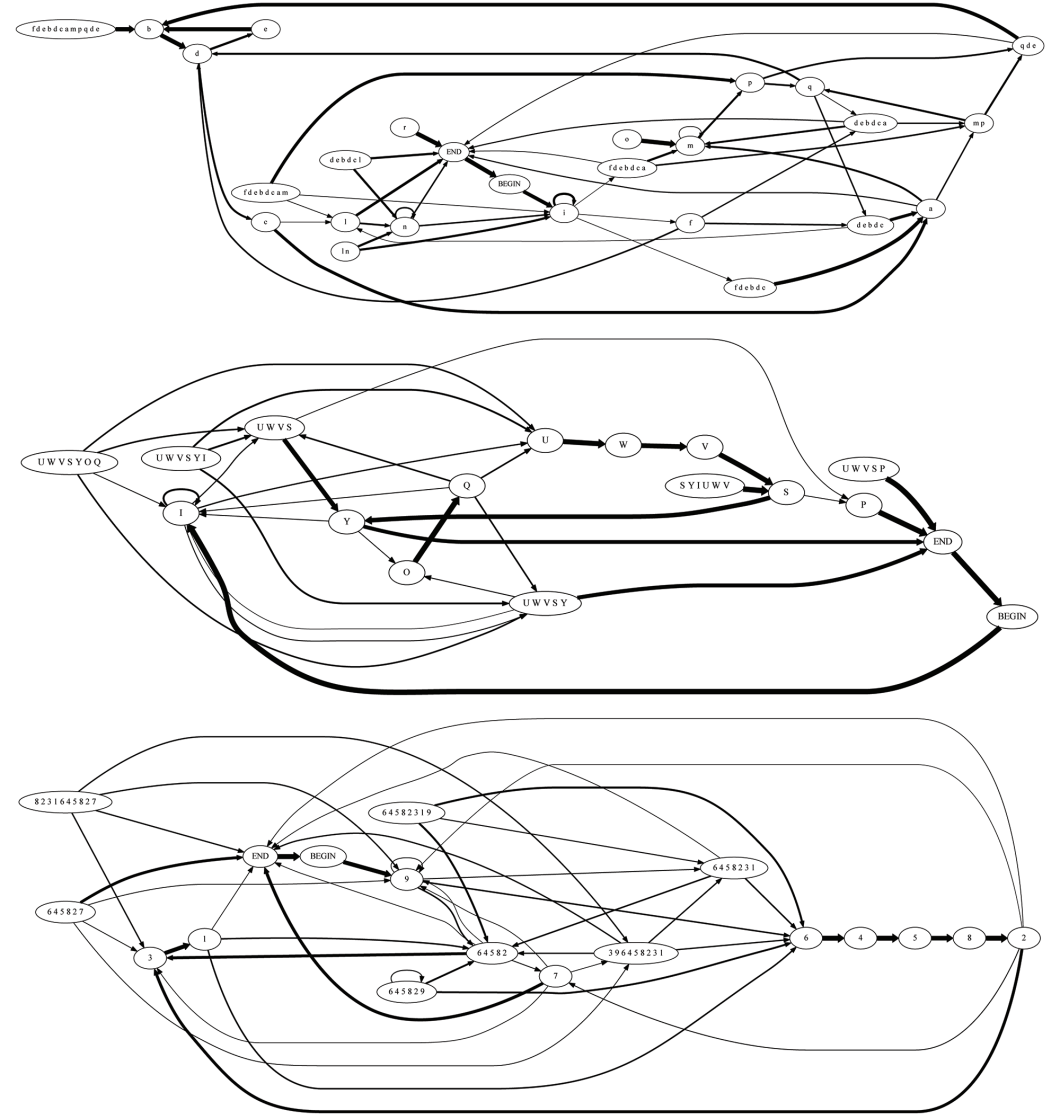
**Examples of grammars of three individuals: these graphs describe the finite-state grammar of type COL without introductory notes, derived for two unrelated juveniles and the father of one of them**. From top to bottom: unrelated juvenile, son, father. Transitions with probability <0.1 were omitted for clarity. To avoid confusion, the syllables in each bird’s song were assigned unique characters. Thus, syllables in the repertoire of individual 625 are denoted by digits, syllables in the repertoire of individual 423 by uppercase letters, and syllables in the repertoire of individual 303 by lowercase letters. Importantly, our analysis does not rely on syllable-level correspondence among individuals. The spectral similarity measure, according to which grammars of fathers and sons are more similar to each other than grammars of unrelated individuals, is too complex and spatially distributed to be visually apparent in a casual inspection of the graphs.

To summarize, we find that grammars of related birds, despite being far from structurally identical, are more similar than grammars of unrelated birds (see example in **Figure 5**). While many avenues for further exploration of the precise structural characteristics of this similarity suggest themselves, the present finding is quite robust: it holds across multiple grammar types and similarity measures, all based on graph-structure alone, with no assumptions regarding repertoire overlap or commensurability – a correspondence in pattern rather than in sound.

## Discussion

The analysis of graph-based distances revealed that related birds sang songs that were more similarly structured to each other compared to songs of birds from different families. We quantified song structure using graph-based methods that did not require a common labeling scheme of specific syllables across individuals. We find that just as humans learn patterns for using words in a language that are distinct from the specific words that they incorporate (Gomez and Gerken, 1999), juvenile males learn the syntactical structure of song from their fathers in addition to the acoustic form of notes and syllables. The basic finding of a higher within-family structural similarity obtains, to varying extents, for a range of grammars used to describe the birds’ song corpora. This is encouraging, as it suggests that this finding is quite robust.

It is important to note that the grammar that was found in our exploration to perform the best in describing the song is one that allows the representation of regularities that are defined over high-order sequences of syllables. To the best of our knowledge, such grammars have not been applied to birdsong so far; this finding suggests that doing so may be fruitful. The particular aspects of the song that such grammars can capture remain to be characterized in future exploration. A telltale finding may be that the COL grammar did better than others when introductory notes were not omitted from the analysis, suggesting that its advantage stems at least in part from its ability to represent a series of successive appearances of a single note hierarchically, as a higher-order sequence.

We believe the present study is the first to compare grammars across multiple individuals in a songbird species in ways that are independent of acoustical sound features, thus overcoming a major obstacle in the study of birdsong. Past research on song learning has focused on the acoustic content of song and the extent of copying; studies of syntax (e.g., Rose et al., 2004, involving white-crowned sparrows) likewise assume a fixed repertoire of syllables shared by all members of the species. With the zebra finch, several laboratories have tried to develop a universal classification system based on note shape, each using slightly different number and type of categories (Zann, 1993; Sturdy et al., 1999; Lachlan et al., 2010). However, even in the zebra finches’ relatively simple song, developing a catalog for every song element across individuals has been difficult. Instead of trying to reconcile individual differences in song elements, our method allows direct comparison of the grammars of individuals without calling for a specific acoustic classification system. Furthermore, our method may be exceptionally well-suited for studying song in bird species with large song repertoires. In such species, song classification based on note types would be particularly difficult, and a grammar-based method correspondingly useful.

Using the full song corpus of each individual, including the introductory notes and variability that so far has been typically dismissed, allowed us to show that song learning in the zebra finch is more than a process of mimicking the father’s syllables and learning their linear canonical order, as has been assumed in the past. This finding is in line with some previous observations: in aviary settings, zebra finches learn hybrid songs composed by elements from multiple tutors, copied as chunks (Williams, 1990; Williams and Staples, 1992). Longer inter-syllable durations, transitions between call- and non-call like elements, and locations of song-breaks in the tutor song mark boundaries of the copied chunks (Williams and Staples, 1992). Our results show that juveniles learn underlying statistical structure of the song, beyond syllable-level correspondence between learner and tutor songs. This ability suggests that the birds use statistical learning mechanisms to map out the hierarchical organization of the tutor’s song into an internally represented grammar of song production. Human adults and prelinguistic infants are sensitive to statistical regularities in segmentation tasks when learning artificial and natural languages (Saffran et al., 1996; Gomez and Gerken, 2000; Onnis et al., 2008; Goldstein et al., 2010). Our present finding of such sensitivity to grammar in songbirds indicates a new parallel between song learning in birds and language learning in humans.

The kind of statistical learning mechanism that allows zebra finches to learn grammatical structure is applicable also to the general problem of learning structured, serially ordered behavior. For instance, statistical learning can be useful in learning the structure of foraging environments, food handling, tool use, and organizing one’s behavior within a complex social group. Though these behaviors transcend domains, species, and scientific disciplines, the computational tools used here are capable of revealing their underlying grammatical structures and yield insight into the cognitive capacities necessary for learning adaptive skills.

## Conflict of Interest Statement

The authors declare that the research was conducted in the absence of any commercial or financial relationships that could be construed as a potential conflict of interest.
